# MUCOCUTANEOUS MANIFESTATIONS OF CHIKUNGUNYA FEVER

**DOI:** 10.4103/0019-5154.60356

**Published:** 2010

**Authors:** Debabrata Bandyopadhyay, Sudip Kumar Ghosh

**Affiliations:** *From the Department of Dermatology, STD, and Leprosy, R. G. Kar Medical College, Kolkata 700 004, India.*

**Keywords:** *Chikungunya*, *mucocutaneous*, *treatment*

## Abstract

Chikungunya fever (CF) is an arboviral acute febrile illness transmitted by the bite of infected *Aedes* mosquitoes. After a quiescence of more than three decades, CF has recently re-emerged as a major public health problem of global scale. CF is characterized by an acute onset of high fever associated with a severe disabling arthritis often accompanied by prominent mucocutaneous manifestations. The disease is usually self-limiting, but the joint symptoms and some of the cutaneous features may persist after the defervescence. A wide range of mucocutaneous changes has been described to occur in association with CF during the current epidemic. Besides a morbilliform erythema, hyperpigmentation, xerosis, excoriated papules, aphthous-like ulcers, vesiculobullous and lichenoid eruptions, and exacerbation of pre-existing or quiescent dermatoses had been observed frequently. These unusual features may help in the clinical differential diagnosis of acute viral exanthems mimicking CF.

## Introduction

Chikungunya fever (CF) is an acute viral illness caused by an arbovirus of the same name transmitted by the bites of *Aedes* mosquitoes. Documented first time from an outbreak in Tanzania in 1952,[1,2] explosive outbreaks of epidemics of the disease have occurred after periods of long quiescence in different parts of the world. After an extensive outbreak during the beginning of the current millennium in the French territory of Reunion Islands in the Indian Ocean, CF has been reported from almost 40 countries from different regions of the world.[3] After an interval of 32 years, India has witnessed a massive epidemic in 2005, which is still ongoing in different parts of the country.[4] The disease has affected millions of people and left many with crippling disabilities.[3]

A human-mosquito-human cycle is responsible for the maintenance of the virus in the South-East Asia region in contrast to the sylvatic transmission cycle occurring in the African continent.[3] The re-emergence of CF has been attributed to a multitude of factors including mutation of the virus, absence of herd immunity, lack of efficient vector control activities, and globalization and emergence of another vector, *A. albopictus*, in addition to *A. aegyptii*, as an efficient transmitter of Chikungunya virus.[5]

CF may affect people of all age groups with an equal gender distribution. After an incubation period ranging from 3 to 12 days, there is usually an abrupt onset of high fever accompanied by severe arthralgias, myalgias, and skin rash. There may be conjunctival suffusion, persistent conjunctivitis, and cervical or sometimes generalized lymphadenopathy, together with swollen and tender joints frequently involving the small joints of the hand, wrist, and ankles, but also involving the larger joints such as knee and shoulder in some patients.[4] Other clinical manifestations of CF may occur in the forms of photophobia, retro-orbital pain, vomiting, diarrhea, and neurological affection such as meningeal syndrome and acute encephalopathy.[3]

While the clinical feature of CF is dominated by the sudden onset of high fever and the disabling joint symptoms, mucocutaneous manifestations occur in a significant proportion of cases. A wide array skin and mucous membrane lesions have recently been documented in literature particularly by Indian workers looking specifically into this aspect of the disease during the current epidemic.[6–9] We have reviewed various dermatological affections of CF in this article.

## Mucocutaneous Manifestations

**Figure 1:**
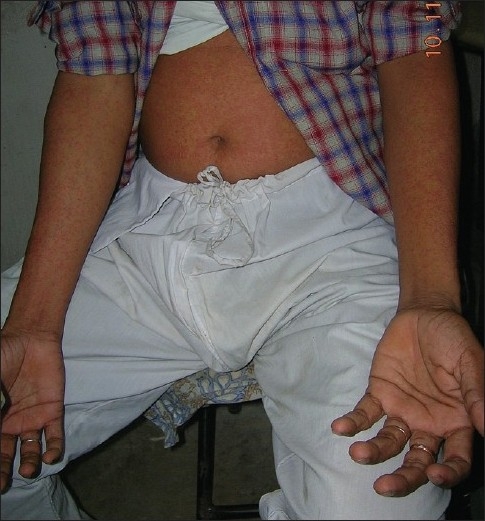
Morbilliform rash developing acutely with the onset of fever

A large variety of skin and mucous membrane lesions have been documented to occur in association with CF including some that have not been described in other viral exanthems [Table 1]. The dermatological manifestations of the disease may occur in about 40-50% of all cases.[10,11] Morbilliform eruption [Figure 1] is the most common pattern of cutaneous lesions found.[6,9] It usually appears 3 to 5 days after the appearance of fever and subsides within 3 to 4 days usually without any sequelae. The rash is asymptomatic in about 80% of the patients, and the remainder may complain of mild pruritus.[6] The eruption most frequently appears on the first 2 days of onset of fever, but may appear simultaneously with the fever or after defervescence.[6] The first site of appearance of the skin lesions are most frequently the upper limbs, followed by the face and trunk.[6] The skin rash in CF commonly affects the extremities, trunk, neck, and ear lobes. Although the face is said to be relatively spared,[4] facial involvement in up to 77% of the cases have been documented.[6] Recurrent episodes have also been observed.

**Table 1:** Mucocutaneous manifestations of Chikungunya fever

Cutaneous changes	
Morbilliform eruption	Lymphedema
Hyperpigmentation	Vasculitic lesions
Xerosis with scaling	Lichenoid eruption
Desquamation of palms	Erythema nodosum
Excoriated papules	Erythema multiform-like lesions
Generalized urticarial lesions	Peripheral cyanosis
Penoscrotal and perineal ulcer	Exacerbation of preexisting dermatoses (psoriasis, lichen planus, melasma)
Generalized erythema	
Transient nasal erythema	
Vesiculobullous lesions	Unmasking of previously undiagnosed leprosy with type I reaction
Ecchymoses	
**Mucosal lesions**	

Aphthous ulceration	
Depigmented macules on lips	
Crusted lesions on the lips and angle of mouth	
Oral mucosal pigmentation	
**Nail changes**	

Subungual hemorrhage	

**Figure 2:**
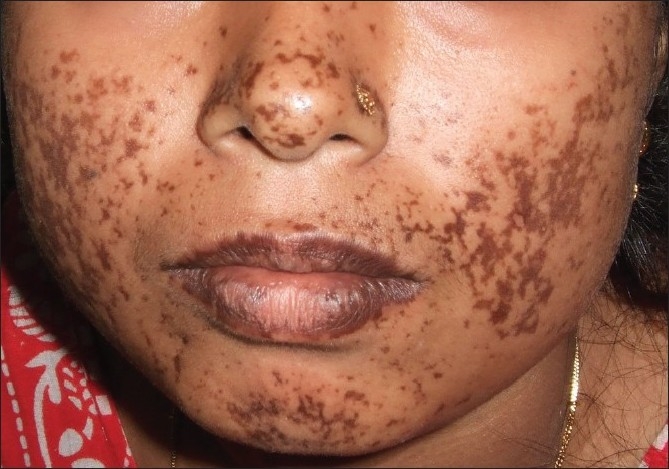
Persistent facial hypermelanosis following Chikungunya fever

In a number of patients, hypermelanosis of the skin may develop soon after the rash has resolved [Figure 2]. The hypermelanosis appears to be postinflammatory in nature and may develop rapidly.[6] The hyperpigmentation may be of different types including centrofacial and freckle-like, diffuse pigmentation of face, pinna, and extremities, flagellate pigmentation, and pigmentation of existing acne lesions.[7] Predominant affection of the exposed skin raises the possibility of the role of ultraviolet exposure in the distribution pattern of the pigmentary anomaly. Xerosis of skin and associated scaling is also commonly seen.[6] Desquamation of palms was also noted in some patients. Excoriated papules due to itching are often present. Generalized urticarial lesions have also been reported to be associated with CF in a number of studies. Generalized macular erythema, usually found within 24-48 h of appearance of fever, is another important finding of this infection.

Acute intertrigo-like lesions and peno-scrotal or perianal ulceration are other distinctive manifestations of CF.[8] The patients usually develop these ulcers about 2-5 weeks after the onset of fever. The ulcerations are usually punched-out, deep-seated with undermined edges showing healthy granulation tissue in the floor and erythema, and thickening in the surrounding skin. The size of the ulcers varies from 0.5 to 2 cm in diameter and their shape is round to be oval or asymmetrical. The number of ulcers per patient usually ranges from 1 to 3.[8] These lesions are self-limiting. Apart from these, multiple aphthous-like ulcers may also be found on axillae, tongue, palate, and other areas of oral mucosa.[8] Nasal erythema may develop after 24-48 h after the appearance of fever. Lymphedema, mainly in acral distribution, may also appear 2-3 weeks after the appearance of fever. Flaccid vesiculobullous lesions in infants have also been reported.[12] These lesions were of sudden-onset and often multiple and healed without scarring or pigmentary changes. Vesiculobullous lesions appeared around the fourth day of fever over the lower limb and spread to involve the perineum, abdomen, chest, and upper limb.[12] Generalized erythema, maculopapular rash, and skin peeling were among the other dermatological findings in the infants. A high incidence of peripheral cyanosis (without any hemodynamic alteration) was observed among the infants.[12]

Vasculitic lesions and erythema nodosum like lesions have also been reported to occur in CF.[9] Targetoid lesions over the extremities and trunk simulating erythema multiforme was seen in some patients.[9] Exacerbation of the pre-existing dermatoses may take place in the setting of CF. Exacerbation of psoriasis in remission,[7,9] unmasking of previously undiagnosed leprosy with type I reaction, and accentuation of melasma,[7] and lichen planus[9] have been documented.

Apart from the aphthous ulcerations and gingivitis,[13] depigmented macules on lips, crusted lesions on the lips and angle of mouth, and oral mucosal pigmentation could be among the other oral mucosal findings. Nail changes occur very rarely. Only a few cases of subungual hemorrhage have been reported in the literature. Delayed cutaneous manifestations (beyond 1 month) may also occur in the form of hyperpigmentation, maculopapular eruption, aphthous-like ulcer, lichenoid eruption, subungual hemorrhage, and lymphedema.[7] In contrast to dengue fever, hemorrhagic manifestations are rare in CF. When present, they are usually mild and more frequently encountered in Asian populations. Multiple petechial lesions and ecchymoses may also accompany the clinical presentation.

## Sequelae

Although the acute febrile illness caused by the Chikungunya virus remits spontaneously without any sequelae in most patients, the joint manifestations may linger for a prolonged period of time. Persistent joint affection has been described to occur in about 12% of patients in the forms of residual stiffness without pain and persistent painful restriction of joint movements.[14] Neurological and emotional sequelae have also been described.[3] As described above, some dermatological manifestations may appear weeks after the other changes have subsided. Hyperpigmentation of skin may persist for months after the remission of CF. Xerosis has also been seen to pursue a prolonged course necessitating regular application of emollients for symptomatic relief.

## Differential Diagnosis

Acute CF requires to be differentiated from a wide range of conditions that present with acute febrile exanthems with arthralgia. The principal Chikungunya mimic is dengue fever, which may occur as a co- infection in association with CF.[15] It is often impossible to distinguish the two on clinical grounds alone and a serological exclusion is desirable. Other conditions that may have to be differentiated by appropriate clinical and laboratory features include rubella, measles, infectious mononucleosis, hepatitis B infection, scarlet fever, Kawasaki disease, toxic shock syndrome, acute retroviral syndrome, malaria, leptospirosis, rheumatic fever, and drug reactions. The chronic arthropathy that may follow acute CF has to be differentiated from other rheumatological disorders like rheumatoid arthritis and systemic lupus erythematosus. The persistent facial hypermelanosis may closely mimic melasma. The oral and genial aphthous-like ulcerations require differentiation from Behcet's disease and ulcerative sexually transmitted infections such as syphilis and chancroid.

## Diagnosis

Diagnosis of CF is based on a high index of suspicion while dealing with any case of acute febrile illness with joint symptoms particularly in the appropriate epidemiologic setting. While a morbilliform rash is common to many viral and bacterial exanthems, the unusual mucocutaneous features associated with CF, such as the rapidly developing facial hypermelanosis, aphthous-like ulcers, and the intense xerosis and scaling, may help in the differential diagnosis. A laboratory confirmation of diagnosis should ideally be done with the appropriate virological and serological tests, but these are rarely available in areas where the outbreak has occurred. Thus, the vast majority of cases are diagnosed on clinical and epidemiological grounds. A positive virus culture supplemented with neutralization provides a definitive proof for the presence of Chikungunya virus.[16] A reverse transcriptase polymerase chain reaction (RT-PCR) test can also provide proof of infection. Demonstration of a fourfold increase in specific IgG antibody titer against the virus between the acute and convalescent phase sera, or the demonstration of IgM antibodies specific for Chikungunya virus in acute-phase sera, offers serological confirmation of the disease.

## Treatment

There is no specific antiviral therapy available for CF. The disease is generally self-limiting and the goal of the therapy is symptomatic relief of complaints like fever and joint paint with paracetamol or NSAIDs. Since no vaccines are commercially available for inducing active immunity against the disease, the mainstays of prevention remain the vector control measures at the household and community levels and the avoidance of mosquito bites by appropriate measures.

The mucocutaneous features are managed with symptomatic treatments like the use of oral antihistamines with or without application of soothing agents like calamine lotions for those patients who complain of pruritus associated with the rash. Xerosis and scaling may be treated with the application of mineral or vegetable oils or other emollients on moistened skin. We have seen facial hypermelanosis to persist for a long time after the remission of the acute febrile episode. This may be treated with topical hypopigmenting agents like hydroquinone with or without short-course topical steroids. Photoprotective measures including the usage of sunscreens should also be advised. Intertriginous and penoscrotal ulcerations may be treated with topical or systemic antibacterials for prevention or treatment of secondary bacterial infections.

## References

[1] Robinson MC (1955). An epidemic of virus disease in Southern Province, Tanganyika Territory, in 1952-53; I. Clinical features. Trans R Soc Trop Med Hyg.

[2] Lumsden WH (1955). An epidemic of virus disease in Southern Province, Tanganyika Territory, in 1952-53; II. General description and epidemiology. Trans R Soc Trop Med Hyg.

[3] World Health Organization, Regional Office for South-East Asia (2008). Guidelines on Clinical Management of Chikungunya Fever.

[4] Mohan A (2006). Chikungunya fever: clinical manifestations and management. Indian J Med Res.

[5] World Health Organization Proposed case definition of chikungunya fever: WHO South East Asia Regional Office.

[6] Bandyopadhyay D, Ghosh SK (2008). Mucocutaneous features of Chikungunya fever: a study from an outbreak in West Bengal, India. Int J Dermatol.

[7] Inamadar AC, Palit A, Sampagavi VV, Raghunath S, Deshmukh NS (2008). Cutaneous manifestations of chikungunya fever: observations made during a recent outbreak in south India. Int J Dermatol.

[8] Mishra K, Rajawat V (2008). Chikungunya-induced genital ulcers. Indian J Dermatol Venereol Leprol.

[9] Prashant S, Kumar AS, Mohammed Basheeruddin DD, Chowdhary TN, Madhu B (2009). Cutaneous manifestations in patients suspected of chikungunya disease. Indian J Dermatol.

[10] Staikowsky F, Talarmin F, Grivard P, Souab A, Schuffenecker I, Le Roux K (2009). Prospective study of Chikungunya virus acute infection in the Island of La Reunion during the 2005-2006 outbreak. PLoS One.

[11] Simon F, Parola P, Grandadam M, Fourcade S, Oliver M, Brouqui P (2007). Chikungunya infection: an emerging rheumatism among travelers returned from Indian Ocean islands. Report of 47 cases. Medicine (Baltimore).

[12] Valamparampil JJ, Chirakkarot S, Letha S, Jayakumar C, Gopinathan KM (2009). Clinical profile of Chikungunya in infants. Indian J Pediatr.

[13] Suryawanshi SD, Dube AH, Khadse RK, Jalgaonkar SV, Sathe PS, Zawar SD (2009). Clinical profile of chikungunya fever in patients in a tertiary care centre in Maharashtra, India. Indian J Med Res.

[14] Kennedy AC, Fleming J, Solomon L (1980). Chikungunya viral arthropathy: a clinical description. J Rheumatol.

[15] Chahar HS, Bharaj P, Dar L, Guleria R, Kabra SK, Broor S (2009). Co-infections with chikungunya virus and dengue virus in Delhi, India. Emerg Infect Dis.

[16] World health organization http://www.searo.who.int/EN/Section10/Section2246_12902.htm.

